# Assessing and managing patients with borderline personality disorder requesting medical assistance in dying

**DOI:** 10.3389/fpsyt.2024.1364621

**Published:** 2024-06-11

**Authors:** Paul S. Links, Hira Aslam, Jonah Brodeur

**Affiliations:** ^1^Department of Psychiatry and Behavioural Neurosciences, McMaster University, Hamilton, ON, Canada; ^2^Michael G. DeGroote School of Medicine, McMaster University, Hamilton, ON, Canada; ^3^Department of Family Medicine, University of Toronto, Toronto, ON, Canada

**Keywords:** borderline personality disorder, assessment, management, irremediability, treatment resistant, medical assistance in dying

## Abstract

**Background:**

When physician assisted dying (referred to as Medical Assistance in Dying or MAiD in this article) is available for individuals with mental disorders as the sole underlying medical condition (MD-SUMC), patients with borderline personality disorder (BPD) frequently request MAiD. Psychiatrists and other clinicians must be prepared to evaluate and manage these requests.

**Objectives:**

The purposes of this paper are to define when patients with BPD should be considered to have an irremediable, treatment resistant disorder and provide clinicians with an approach to assess and manage their patients with BPD making requests for MAiD.

**Methods:**

This perspective paper developed the authors’ viewpoint by using a published, authoritative definition of irremediability and including noteworthy systematic and/or meta-analytic reviews related to the assessment of irremediability.

**Results:**

The clinician must be aware of the eligibility requirements for granting MAiD in their jurisdiction so that they can appropriately prepare themselves and their patients for the assessment process. The appraisal of the intolerability of the specific person’s suffering comes from having an extensive dialogue with the patient; however, the assessment of whether the patient has irremediable BPD should be more objectively and reliably determined. A systematic approach to the assessment of irremediability of BPD is reviewed in the context of the disorder’s severity, treatment resistance and irreversibility.

**Conclusion:**

In addition to characterizing irremediability, this paper also addresses the evaluation and management of suicide risk for patients with BPD undergoing the MAiD assessment process.

## 1 Introduction

In the near future, Canada will join countries such as Luxembourg, Belgium, The Netherlands, and Switzerland (*allows assisted suicide not physician assisted death)* in which Medical Assistance in Dying (MAiD) is available for individuals with mental disorders as the sole underlying medical condition (MD-SUMC) (1). MAiD has been available in Canada since June 2016; however, the extension of eligibility to include persons with mental disorders has been delayed until 2027 particularly because of the difficulties in defining when mental disorders are irremediable (see for background to MAiD in Canada - https://www.canada.ca/en/health-canada/services/health-services-benefits/medical-assistance-dying/legislation-canada.html) (2).

In countries where people with mental disorders are eligible for MAiD, research has identified that the proportion of patients requesting MAiD due to borderline personality disorder (BPD) was as high as 27% (3). A physician survey from the Netherlands identified that among patients requesting MAiD, 64% were diagnosed with personality disorders (4, 5). When MAiD becomes available to patients with BPD, psychiatrists and other clinicians have to be prepared to evaluate and manage these requests. Although patients with mental disorders may not be eligible for MAiD until 2027 in Canada, issues related to personality disorders are still evident in other MAiD assessments. Weibe and Kelly (6) surveyed MAiD providers assessing patients whose death was not reasonably foreseeable but had grievous and irremediable medical conditions that caused unbearable suffering and they noted that amongst these patients “Personality disorder very present and make patient interactions challenging”

To enrich the discussion, the following simulated case vignette is provided of a patient with BPD making a request for MAiD:

*Mr. A is a 39-year-old single man who is followed by his psychiatrist and primary care team for major depressive disorder, social anxiety disorder and BPD. Mr. A has a history of previous admissions for serious suicide attempts including attempted hanging. He has been inconsistently employed since dropping out of college when a co-student threatened to charge him with sexual harassment. He is now reliant on disability. On one previous admission, the possibility that the patient had a comorbid diagnosis of Autism Spectrum Disorder was raised but never confirmed. After many treatment attempts, the patient is fixated that he has an untreatable disorder and he insists he wants an assessment when MAiD-MD SUMC becomes available.*

Our purposes are to define when patients with BPD should be considered to have an irremediable, treatment resistant disorder and provide clinicians with an approach to assess and manage their patients with BPD making requests for MAiD using the four phases of the MAiD process as outlined by the Dutch Psychiatric Association guideline (1):

1. Request phase.
2. Assessment phase – in Canada, this phase includes consultation with an expert regarding the relevant diagnosis.
3. Consultation phase – awareness of the psychological and social factors behind the request.
4. Implementation of the assessment decision phase – or care after refusal.

The emphasis of this report is on the assessment phase and BPD irremediability given the paucity of information on how to make such determinations. This paper also addresses the management of suicide for patients with BPD undergoing the MAiD assessment process.

## 2 Methodology

This perspective is written to provide the authors’ viewpoint on a relevant clinical subject so a formal review methodology was not employed. However, the authors adopted the following approach in developing the paper:

1) A published, authoritative definition of irremediability was utilized (see section 3.1) and
2) For each topic related to the assessment of irremediability (severity, treatment resistance, irreversibility), noteworthy systematic and/or meta-analytic reviews were identified by searching Google Scholar for the last decade and included.

## 3 Request phase

When the patient with BPD expresses a wish for MAiD, the physician must create an open and safe atmosphere for dialogue, carry out an assessment for acute suicidality, check whether relatives are aware of the patient’s request, provide accurate information about the assessment process and, if appropriate, provide an effective referral. This means taking positive, timely action to connect the patient to the right services or professionals. Involving relatives and family will vary from one person to another. Verhofstadt et al. (7) noted that some individuals assessed for MAiD based on psychiatric conditions reported valuing some relatives being involved while others raised concerns and strongly opposed involvement. Although the issue of excluding families is not unique to persons with BPD, clinicians should be aware that adverse childhood experiences are more common in patients with BPD (8). Family members exclusion should be comprehended with this knowledge as they may be perpetrators of the abuse.

When patients with BPD request MAiD, clinicians must be prepared to evaluate and differentiate these requests from suicidal crises. Suicidal crises and MAiD requests can both be driven by patients’ psychological suffering and perception that they are a burden on others (3, 4). However, suicidal crises as characterized by Schneidman (9) tend to be more acute, more impulsive and their psychological state may render the patient incapable. The wish to die for patients requesting MAiD should be ongoing, reflective, and considered with full capacity. Patients in an acute suicidal crisis would not be candidates for MAiD and should receive typical interventions to prevent suicide (10).

The MAiD assessment process and managing the risk of suicide is not unique to patients with BPD but an empathic assessor may have therapeutic impacts on these patients. Kammeraat et al. (11) reported that 72% of patients who withdrew their requests for euthanasia or assisted suicide had a diagnosis of personality disorders and they noted that validation of their suffering seemed to help patients with personality disorders cope better with their suffering and realize they do not wish to die.

## 4 Assessment phase

Clinicians must be aware of the eligibility requirements for granting MAiD in their jurisdiction so that they can appropriately prepare patients for the assessment process.

In Canada, like other jurisdictions, when assessing persons’ eligibility for MAiD, they must have a grievous and irremediable medical condition. The condition must cause “enduring physical or psychological suffering that is intolerable to them and that cannot be relieved under conditions that they consider acceptable” (12). Patients with BPD endorse significant suffering, are characterized by an elevated lifetime risk of suicide as high as 10%, have frequent histories of suicide attempts and recurrent suicide crises. These features support the existence of significant suffering for these patients but clinicians can only appraise the intolerability of the specific person’s suffering after extensive dialogue with the patient (3).

However, the assessment of whether the patient has irremediable BPD should be objectively and reliably determined to ensure that eligibility decisions are made equitably and fairly. Clinicians should be knowledgeable about and inform their patient that the following may be considered during the assessment for irremediable BPD:

### 4.1 Defining irremediable

Irremediability can be defined as an inability to sufficiently reduce the patient’s symptoms and/or suffering as the term *incurable* is not typically applied to mental disorders (3). Our position on irremediability in the context of MAiD evaluations is a functional rather than an ontological definition, driven by the necessity of developing a framework for assessment rather than a statement about the existence of irremediability in mental disorder itself. The Dutch and Flemish Psychiatry Associations have published advisory or guidance documents on assessing irremediability (13–15). In addition, the Council of Canadian Academies, The State of Knowledge for MAiD Mental Disorder is the Sole Underlying Medical Condition indicated that several other factors need to be considered when defining incurable conditions (16).

First, a mental disorder may be considered *chronic* and, therefore, the focus of treatment maybe on restoring function rather than a cure (16). Second, the disorder may be *treatment resistant*, defined as the inability to meaningfully reduce symptoms after a certain number, or type, of interventions (16). Third, a mental disorder may also be considered incurable if the available treatments are considered *unacceptable* to the patient and there is a shared determination of the remaining treatments by the clinician and the patient (17). Lastly, the ability to *reliably judge* irremediability remains difficult to ascertain for mental disorders but this process continues to be refined (16).

All of the above considerations (chronicity, treatment resistance, acceptability and reliability) may be considered when assessing patients with BPD requesting MAiD. Van Veen et al. (1) found that almost 90% of MAiD requests due to psychiatric suffering did not end in physician assisted death and patients with Cluster B personality disorders were frequently rejected. Therefore, clinicians and patients should be aware of the issues that arise during the MAiD assessment to determine if a patient has irremediable BPD (10).

### 4.2 Assessment of irremediability

#### 4.2.1 Severity

The assessment may start by determining the severity of the mental illness at the current time and based on the evolution of the condition to that point in time (10). Moran and Crawford attempted to defined BPD severity but felt that there was no agreed upon measure of severity (18). However, the Alternative Model for Personality Disorders Criterion A and the ICD 11 definition of personality disorders have focused on the issue of severity and have provided an approach to assessing the severity of personality disorders (19, 20). A global evaluation of personality severity requires an enduring disturbance in functioning of aspects of self (e.g. identity, self-worth, accuracy of self-view, self-direction), and/or interpersonal functioning (e.g. ability to develop and maintain close and mutually satisfying relationships, ability to understand others’ perspectives and to manage conflict in relationships) (20). ICD 11 reflects the evidence that severity, rather than type, of personality pathology is the major predictor of suffering and dysfunction and that the total number of diagnosed personality disorders or the number of traits explains more variability in functioning than *a* specific personality diagnosis alone (21). Research suggests that the BPD diagnosis appears closely linked to the Alternative Model for Personality Disorders Criterion A, thus supporting a focus on the level of severity of global personality functioning (21). The level of severity of personality functioning can be assessed systematically by using measures like the Levels of Personality Functioning Scale-Self Report (LPRS-SR) (22). This self-report measure has high internal consistency, significant concurrent validity with other measures of global level of dysfunction and is determined to reflect a single dimension of personality dysfunction (22). The measure has no validity scale regarding the accuracy of the self-report; therefore, collateral history remains important to obtain. Although other measures of personality dysfunction exist, the LPRS-SR has the benefit of being freely available.

#### 4.2.2 Establishing incurability/treatment resistance (from recommendation 2)

The assessment will undoubtedly include reference to patients’ previous treatments and their outcomes (23). Although this assessment must be done on a case-by-case basis, “the incurability of a mental disorder cannot be established in the absence of multiple attempts at interventions with therapeutic aims” (page 13) (10). Mehlum et al. recommended adherence to guidelines for the state-of-the-art treatment for BPD as a measure of the adequacy of previous treatments (24). In Canada, no such guidelines exist regarding BPD, however our systematic review of available guidelines in the English language provides some consensus that structured psychological therapies for a year rather than three months or less specifically developed for BPD are the first line interventions. The guidelines noted that patients can require longer treatment periods (25). Patients with BPD response to psychotherapy is still poorly understood and robust predictors of response have not been established. Woodbridge et al. carried out a systematic review of non-response to psychotherapy using a definition of treatment response as reaching symptomatic remission from the diagnosis BPD or reaching criteria for reliable or clinically significant change indices. Using this definition, the mean rate across studies of non-response was 48.80% (SD = 22.77) (26). This review suggested that generalist models had similar remission rates to specialist treatments. However, Rameckers et al. carried out another meta-analysis including pre- to post-treatment data from all treatment studies (randomized controlled trials (RCT) and non-RCT designs) and outcome domains found the opposite - that is, specialized therapies such as Schema Therapy, Mentalization-based Treatment and reduced (without all components) Dialectical Based Therapy (DBT) were associated with relatively larger effect sizes compared to the average of all treatments (27). Nicolini et al. (28) studied a case series from Dutch regional euthanasia review committee’s cases involving personality and related disorders from 2011 to 2017, where two thirds of cases mentioned cluster B personality disorders or traits. They found that psychotherapy had been tried in 72% of cases but mostly of an unspecified nature. In Canada, like in many countries, the access to treatments is limited and, in many settings, the only available evidence-based therapy is DBT. As a result, assessors may have to consider whether an intervention had the characteristics of effective psychological treatments for BPD as reported by published consensus documents that distilled these characteristics from evidence-based therapies or existing guidelines (see **Table 1**) (29–33).

**Table 1:** Characteristics of Effective Psychological Treatment of BPD.

Consensus with Schiavone and Links, 2013 (29)	Relational Model – Project Air (30)	Clinical practice guideline for the management of borderline personality disorder (31)*	Bateman and Fonagy, 2000 (32)	Weinberg et al., 2010 (33)
Construction of a coherent treatment model				
Therapist has access to supervision				
Improve patient’s ability to connect actions and feelings				
Therapy uses validation alongside change interventions				
Therapist takes an active therapeutic stance				
Accurately gauge potential intensity and lethality of suicidal ideation				
Two tools of active therapists: structured treatment frame and strong attachment relationship				

*Publication was rescinded by the National Health and Medical Research Council, Australian Government.

Pharmacotherapy is not recommended as primary treatment for BPD based on our review of the existing English guidelines (25). However, consideration could be given to management of specific symptoms or co-morbid psychiatric disorders that may be impacting the person’s prognosis or contributing to their suffering (34). When considering interventions for comorbid disorders, findings from prospective longitudinal research of the associations between BPD and Axis I (clinical) disorders must be considered. Although many Axis I disorders are found to be comorbid with BPD, mood and anxiety disorders appear most interrelated. For example, remissions of BPD predict the remission of comorbid major depressive disorder and lessen the risk of future major depressive episodes in patients with BPD; thus, treatment interventions for BPD psychopathology must be undertaken to address this depression and anxiety comorbidity (34). Substance use disorders (SUD) represent a recurrent and intermittent problem among patients with BPD; however, the onset of new SUDs over the course of BPD is uncommon (34). The absence or the remission from a comorbid SUD seems to positively impact the early course of BPD, perhaps over the first 5–7 years (34). Finally, eating disorders are often comorbid with BPD; however, they appear more independent of each other and comorbid patients may require separate interventions for each diagnosis (34).

The assessment may have to clarify the patient’s comorbid disorders; for example, with Mr. A, differentiating severe social anxiety from autism spectrum disorder. Also, consideration must be given to comorbid physical disorders causing pain and suffering and contributing to patients requesting MAiD. A recent systematic review, updating previous reviews (35, 36), identified the frequent comorbidity of sleep disorders, obesity, chronic pain, cardiovascular disease, cerebrovascular disease with BPD (37). While the proportion of patients with BPD that are affected is unclear, the lower life expectancy in patients with personality disorders (38) suggests that, in itself, the presence of BPD can confer vulnerability to morbidity by virtue of impaired physical health, for which treatment should also be offered prior to determining incurability. Indeed, as it remains unknown whether such medical ailments contribute in any way to the pathophysiology of BPD, lack of their treatment prevents assertion that BPD itself is incurable.

#### 4.2.3 Establishing irreversibility/course

Clinicians assessing irreversibility of a mental disorder such as BPD “must come to a shared understanding that the person is in an advanced state of irreversible decline in capacity” (page 13) (10). To make this assessment, the assessor must be knowledgeable about the latest evidence on the course and prognosis of BPD. For example, a recent meta-analysis regarding the course of BPD suggested that between 50–70% of patients with BPD will achieve symptomatic remission at some point between five and fifteen years of follow up (39). Van Veen et al. (23) on assessing irremediability assert that course of the condition was important, stating “If treatment history forms the basis of the decision on irremediability, but relatively little treatment has been possible owing to the fact that the patient has not had symptoms for long, it is reasonable to postpone judgment on irremediability.” Therefore, patients with BPD may be unlikely to be considered eligible for MAiD early in their course.

Although predictors of treatment response and robust prognostic indicators are yet to be established, some prognostic factors have been implicated. Videler et al. summarized the course of BPD as symptoms may wax and wane over time; however, acute symptoms change more rapidly and readily (e.g. suicidality, self-harm) than temperamental symptoms (e.g. dysphoria, feelings of emptiness, fears of abandonment) (40). They reported that predictors of good outcome appear mostly related to personal capacity and competence such as higher IQ, prior good vocational functioning, higher levels of extraversion, agreeableness and lower levels of neuroticism (40). Predictors of poorer outcome related to greater severity and chronicity of the disorder, higher degrees of comorbidity, and a history of childhood adversity (40).

### 4.3 Assessing patient’s decision making

The assessors of patients with BPD need to focus on the patient’s decision making rather than issues of capacity. Doernberg et al. (41) reviewed the capacity assessments from the case summaries published by the Dutch Regional Euthanasia Review Committee but did not examine cases of personality disorders because the authors concluded that “it is more likely that such persons make unwise yet competent choices, and the primary clinical task in such situations is to help patients in their decision-making”. Calati et al. discussed that psychiatric patients requesting MAiD must have intact decision making but also that psychopathology may influence decision making even though it does not mean the patient lacks capacity (5). The Final Report on Expert Panel on MAiD and Mental Illness from Health Canada takes a similar stand on decision making and treatment refusal – that is, a capable refusal of treatments with a favorable benefit/burden balance will not lead to automatic access to MAiD (10). Treatment refusal is common; for example, van Veen et al. (1) found that 56% of Dutch patients who received MAiD due to psychiatric suffering refused some sort of treatment. LeGris demonstrated that decision making in patients with BPD appears to be more impacted by emotional reactivity or instability as “rash decisions are made under the contexts of negative affect” (page 5) (42). If a patient with BPD had access to a psychotherapeutic treatment option that includes the effective characteristics of psychological treatments but was refusing this option because of their current emotional state then eligibility may be questioned. Also, with regards to whether a person understands his/her condition to be irreversible, patients with BPD’s decision making can be influenced by a preference for immediate over delayed rewards (43). The assessor must understand what causes patients with BPD to perceive their suffering as enduring. The person’s interpretations of the persistence and permanence of their suffering resulting in the request for MAiD should reflect a realistic appraisal of their situation rather than result from impaired decision making because of a preference for an immediate reward.

## 5 Consultation phase - awareness of the psychological and social factors behind the request

Clinicians and assessors for patients with BPD requesting MAiD must be able to formulate a psychological understanding of the forces behind the request (5). Transference and countertransference reactions are common when working with patients with BPD and the clinician’s countertransferential guilt, hatred or helplessness could influence the patient’s request and desire for MAiD (44). For example, Mr. A’s psychiatrist may have adopted the patient’s hopelessness and has resigned to his request for MAiD. A careful formulation of the psychological mechanisms when a therapeutic impasse is influencing the request for MAiD may suggest other care pathways rather than MAiD. Calati et al. in their systematic review of euthanasia and assisted suicide in psychiatric patients found that a formulation of the influence of transference-countertransference was infrequently done (5). In addition, clinicians must understand that social determinants and lack of access to evidence-based, trauma-informed and culturally safe mental health care can drive requests for MAiD in patients with BPD (6).

## 6 Implementation of the assessment decision phase – or care after refusal: assessing and managing the risk for suicide

Clinicians should recognize that MAiD assessments for patients with BPD may precipitate a suicide crisis during the assessment or when denied MAiD (1, 45). Isenberg-Grzeda E et al. documented that suicide attempts may be precipitated when patients are determined ineligible. They identified several risk factors that are particularly relevant to patients with BPD including: a history of previous suicide behavior, evidence of hopelessness, rigid and inflexible thought processes, and the patient holding unrealistic expectations about being eligible for MAiD (45).

Verhofstadt et al. (7) found from their qualitative study of adults with psychiatric conditions regarding their experience of the euthanasia assessment procedure reported continuing suicidality. Regardless of the status of their request, some individuals kept suicide as a Plan B for themselves if their requests were refused. For others, suicide remained their plan A because of growing disbelief that MAiD was a dignified, empowering means of dying. The clinician for patients with BPD requesting MAiD must continue to monitor the patients’ risk during and following the assessment process. Isenberg-Grzeda et al. provided useful recommendations for managing MAiD requests for patients at risk for suicide. Several of their recommendations are particularly relevant when managing patients with BPD (45). As indicated, the clinician must understand and manage the patient’s expectations about the MAiD assessment process. Continuity of care should be prioritized by ensuring the patient has a safety plan and providing warm hand-offs between the referring clinician and the assessors. If the assessor has taken a two-track approach (7) meaning besides assessing for eligibility for MAiD has suggested care approaches to alleviate suffering then the clinician should support the patient’s hope for the future by reinforcing the new care pathways or reminding ineligible patients their eligibility could be re-visited in the future.

## 7 Conclusions and next steps

When MAiD is available to persons with MD-SUMC, patients with BPD will likely constitute a significant proportion of individuals requesting MAiD. Clinicians must be informed about the assessments for eligibility and how to manage patients effectively and safely during and after the assessment process. This perspective is provided to clarify functional definitions of irremediability or treatment-resistance in the context of MAiD for patients with BPD. Our intention is to guide clinicians working with patients with BPD requesting MAiD, and hope that future research may test, revise, and refine these recommendations.

## Data availability statement

The original contributions presented in the study are included in the article/supplementary material, further inquiries can be directed to the corresponding author.

## Author contributions

PL: Conceptualization, Writing – original draft, Writing – review & editing. HA: Conceptualization, Writing – original draft, Writing – review & editing. JB: Conceptualization, Writing – original draft, Writing – review & editing.
